# Artificial intelligence-enabled early intracranial volumetric response predicts systemic progression-free survival in the phase III CROWN study

**DOI:** 10.1093/noajnl/vdag236

**Published:** 2026-09-07

**Authors:** Shao-Lun Lu, Yu-Cheng Chang, Chih-Hung Liang, Po-Lin Chiang, Kevin Maresca, Erqi Pollom, Keith Wilner, Jen-Tang Lu, Francesca Toffalorio, Giuseppe Giaccone, Chong Duan

**Affiliations:** Department of Radiation Oncology, National Taiwan University Cancer Center, Taipei, Taiwan; Graduate Institute of Oncology, National Taiwan University College of Medicine, Taipei, Taiwan; Vysioneer Inc., Cambridge, Massachusetts, USA; Vysioneer Inc., Cambridge, Massachusetts, USA; Vysioneer Inc., Cambridge, Massachusetts, USA; Pfizer, Cambridge, Massachusetts, USA; Department of Radiation Oncology, Stanford University, Palo Alto, California, USA; Pfizer, San Diego, California, USA; Vysioneer Inc., Cambridge, Massachusetts, USA; Pfizer, Milan, Italy; Meyer Cancer Center, Weill Cornell Medicine, New York, New York, USA; Pfizer, Cambridge, Massachusetts, USA

**Keywords:** ALK-positive NSCLC, artificial intelligence, early tumor response, lesion-level assessment

## Abstract

**Background:**

Manual endpoint assessment is labor-intensive and variable. This study evaluated artificial intelligence (AI)-powered intracranial response assessment and explored imaging prognostic factors in the phase III CROWN trial of advanced treatment-naive anaplastic lymphoma kinase (ALK)-positive non-small cell lung cancer (NSCLC).

**Methods:**

CROWN randomized patients to lorlatinib or crizotinib. Seventy-two with baseline brain metastases (BMs) had brain MRI every 8 weeks. We evaluated an FDA-cleared AI platform, VBrain, for automated lesion-level tracking of BMs and early tumor response (ETR) in relation to systemic progression-free survival (PFS), the primary endpoint of the trial. Progression-free survival was assessed by blinded central review, independent of AI. VBrain assessments were compared with 2 neuroradiologists (R1, R2). Early tumor response at the first on-treatment scan was derived from modified response evaluation criteria in solid tumors (mRECIST) target-lesion diameters and from AI-based all-lesion volumes (AI-ETR). Treatment-neutral Cox models related ETR to systemic PFS; discrimination was assessed using Uno’s C and time-dependent AUC(*t*) with bootstrap uncertainty.

**Results:**

Per mRECIST v1.1, VBrain, R1, and R2 measured 68, 32, and 51 targets, with high concordance (r = 0.94). Across 666 time-points, pairwise assessment discordance and time to intracranial progression did not differ between AI and readers. Beyond mRECIST, VBrain tracked 322 tumors. AI-ETR significantly stratified PFS (multivariable-adjusted hazard ratio [HR] 0.44, 95% CI, 0.23-0.86), whereas target-diameter ETR was not significant (adjusted HR 0.64, 95% CI, 0.29-1.41). Adding AI-ETR improved prediction vs a clinical base model (Δ*c*-index +0.07; AUC(*t*) 0.71 vs 0.55).

**Conclusions:**

The AI and human readers reached a high agreement in endpoint evaluations. Volumetric all-BMs ETR outperforming target-only mRECIST offers early prognostic information in advanced ALK-positive NSCLC.

**Trial Registration:**

ClinicalTrials.gov identifier: NCT03052608.

Key Points

•Artificial intelligence-powered lesion-level analysis of brain metastases (BMs) to predict progression-free survival in a phase III trial.
•Artificial intelligence-powered volumetric response outperforms response evaluation criteria in solid tumors in anaplastic lymphoma kinase + non-small cell lung cancer (hazard ratio 0.44, *P* = .0167).
•Artificial intelligence shows high concordance with experts in BM response assessment.

Importance of the Study

This is the first lesion-level artificial intelligence (AI) analysis of brain metastases (BMs) to predict progression-free survival (PFS) in a global phase III trial. Using data from the CROWN study in advanced anaplastic lymphoma kinase-positive non-small cell lung cancer, an FDA-cleared AI platform showed strong prognostic value by evaluating volumetric early tumor response (ETR) across all intracranial lesions. Compared with response evaluation criteria in solid tumors-based target-lesion sums, AI-derived volumetric ETR at week 8 correlated more strongly with systemic PFS, offering earlier insights into treatment effect. Artificial intelligence measurements also showed high concordance with blinded independent central review (BICR) across 666 MRI time points. This study supports the integration of AI in clinical trial imaging and demonstrates a scalable tool for early efficacy assessment in BM. These findings lay the groundwork for AI-assisted BICR and support volumetric intracranial response as a novel early endpoint in neuro-oncology drug development.

Lung cancer remains the leading cause of cancer death worldwide,^1^ with approximately 5% of non-small cell lung cancer (NSCLC) harboring anaplastic lymphoma kinase (ALK) rearrangement.^2^ Anaplastic lymphoma kinase-positive NSCLC tends to occur in younger patients and can be aggressive, with a high propensity to develop brain metastases (BMs), in 25%-40% within 2 years.^3^ The advent of ALK tyrosine kinase inhibitors (TKIs) has dramatically improved outcomes for this population. In particular, third-generation ALK inhibitors such as lorlatinib have achieved unprecedented efficacy in the first-line setting. The phase III CROWN trial compared first-line lorlatinib against crizotinib in advanced ALK-positive NSCLC and demonstrated a dramatic improvement in treatment response and prolonged disease control. At the interim analysis, 12-month progression-free survival (PFS) was 78% with lorlatinib vs 39% with crizotinib (hazard ratio [HR] ∼ 0.28, P < .001).^4^ Lorlatinib also produced a notably superior intracranial response among patients with baseline BMs.^4^^,^^5^ With longer follow-up, CROWN reported the longest PFS currently observed in advanced NSCLC, with a 5-year PFS of 60% on lorlatinib vs 8% on crizotinib.^6^ These results underscore the profound prognostic impact of modern ALK inhibitors.

Despite major gains with ALK TKIs, not all patients sustain benefit; in CROWN’s 5-year outcomes, the majority of lorlatinib progressions occurred within the first 2 years, with far fewer events between 3 years and 5 years.^6^ Identifying predictors of long-term remission vs early progression is critical to further personalize therapy and surveillance. Traditional radiologic response assessment by response evaluation criteria in solid tumors (RECIST) provides a standardized but coarse, sum-of-diameters categories that can miss lesion-level heterogeneity. Refined metrics, such as early tumor shrinkage, time-to-response, or depth of response, may better correlate with long-term outcomes in various solid tumors.^7^ In ALK-positive NSCLC, where targeted therapy often yields dramatic tumor regression, the pattern and extent of early lesion response might hold prognostic significance. However, assessment at the lesion level, evaluating tumor burden for all measurable lesions individually rather than as an aggregated sum of selected target lesions, is labor-intensive and subject to interobserver variability in a manual workflow. Therefore, more sophisticated approaches are needed to capture lesion-level response patterns.

Recent advances in artificial intelligence (AI) offer promising tools to address this challenge. Automated segmentation and longitudinal tracking yield consistent, lesion-level quantification across serial scans.^8^ Deep learning models, particularly convolutional neural networks, can extract complex imaging features without manual feature engineering. In lung cancer, deep learning models integrating serial CT scans have shown the ability to significantly predict survival outcomes, with accuracy improving as additional follow-up scans are incorporated.^9^ A segmentation-based prognostic model also stratified NSCLC recurrence and survival risk, outperforming RECIST for overall and disease-specific survival.^10^ These findings highlight the potential of AI-driven analysis of longitudinal imaging data to uncover predictive patterns that may be missed by human assessment.

The current analysis explored the feasibility of AI-automated tumor response assessment and an AI-based mining of lesion-level imaging data from the CROWN trial to predict prognosis in ALK-positive NSCLC patients. We hypothesize that AI cannot only generate response assessments comparable to those in conventional clinical trials but also leverage rich lesion-level data to identify early imaging biomarkers of treatment benefit or failure in advanced ALK-positive NSCLC. Importantly, this study does not seek to validate the AI algorithm itself, but rather to test whether AI-derived quantitative metrics, beyond conventional RECIST, can better capture early tumor response dynamics associated with the clinically meaningful endpoint of PFS. In doing so, this work builds upon the paradigm-shifting success of lorlatinib in CROWN to refine prognostication and identify patients with durable benefits vs early progression.

## Methods

### Data Source and Patients

The CROWN trial (ClinicalTrials.gov identifier: NCT03052608, registration date: February 14, 2017) is an ongoing phase III randomized study assessing patients with locally advanced or metastatic ALK-positive NSCLC, confirmed using the Ventana ALK (D5F3) companion diagnostic immunohistochemical assay. Study design, eligibility criteria, and primary findings have been previously published.^4^^,^^11^ The protocol and amendments were approved by the institutional review board or independent ethics committee at each participating site and were conducted in accordance with the Declaration of Helsinki, Good Clinical Practice guidelines and local laws. All participants provided written informed consent. In summary, patients were randomized in a 1:1 ratio to receive either lorlatinib (100 mg orally once daily) or crizotinib (250 mg orally twice daily). Tumor assessments were performed at screening and then every 8 weeks (±1 week) from randomization until disease progression, as determined by independent RECIST-defined criteria. Imaging evaluations included computed tomography or MRI of the chest, abdomen, and pelvis. Brain MRI was mandatory and required at baseline and at each subsequent tumor assessment, regardless of the patient’s baseline CNS status. Intracranial response was evaluated by an independent review committee using modified RECIST (mRECIST) v1.1 criteria.^12^

### Imaging and Response Quantification

**Figure 1.**
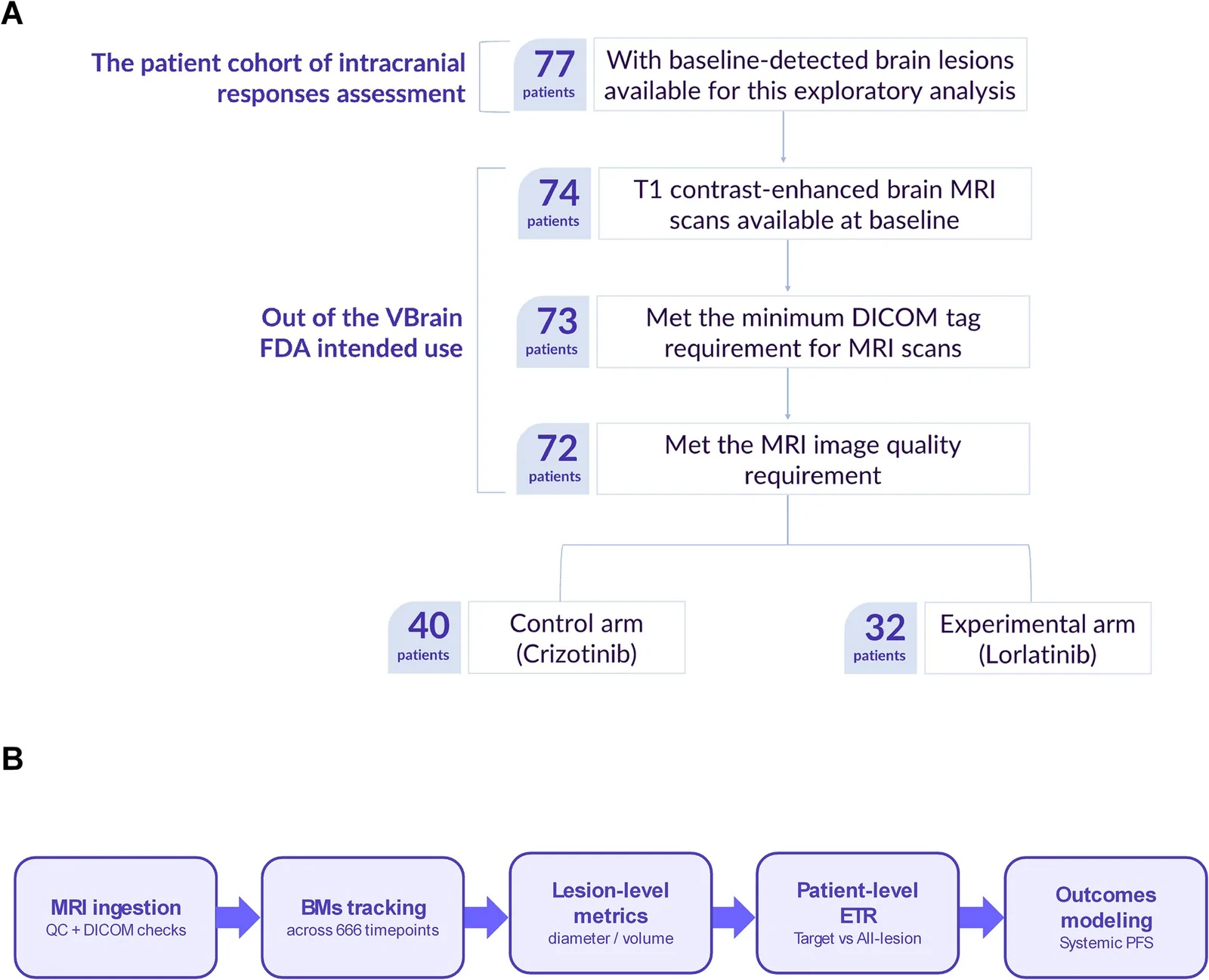
Cohort flow and study pipeline. (A) Screening and eligibility for the intracranial analysis set. Of 77 patients with baseline-detected brain lesions, 74 had contrast-enhanced T1-weighted brain MRI at baseline; 73 met the minimum DICOM-tag requirements; 72 passed image-quality review and formed the analysis cohort (40 in the crizotinib arm and 32 in the lorlatinib arm). Patients excluded during screening were considered outside the VBrain FDA-approved intended use criteria. (B) Study pipeline. Baseline and on-treatment MRIs were ingested with QC/DICOM checks; lesions were automatically and longitudinally tracked to derive per-lesion diameter and volume. Patient-level early tumor response (ETR) was computed at the first on-treatment scan (OTS1) per mRECIST target-lesion diameter and AI-derived all-lesion intracranial volume. The intracranial metrics were correlated with fully independent, adjudicated progression-free survival (PFS) of the CROWN trial.

Intracranial tumors were autosegmented, registered, and tracked for response assessment using VBrain, an FDA-cleared AI platform for brain tumor management.^13^ In this post hoc analysis of the CROWN trial, 77 patients with baseline BMs, regardless of treatment arms, underwent intracranial response assessment using VBrain. Of these, 74 patients had available baseline T1 contrast-enhanced brain MRIs, 73 satisfied the minimum DICOM tag requirements, and 72 met MRI image-quality criteria and entered the analytic cohort (40 crizotinib; 32 lorlatinib; Figure 1).

Early tumor response (ETR) of BMs was assessed at the first on-treatment scan (OTS1, ie the first postbaseline imaging assessment) as the sum of tumor burden relative to the baseline. Figure S1 demonstrates the trajectory of tumor response over time. For each participant, we computed 2 ETR measures (negative values indicate shrinkage): target-lesion ETR: percent change in the sum of target-lesion longest diameters per mRECIST v1.1, and AI all-lesion ETR: percent change in total intracranial tumor volume computed by VBrain across all measurable lesions. For binary analyses, cohort-median cut points were prespecified as −24.2% for RECIST target-lesion diameter and −67.9% for AI all-lesion volume. These metric-specific cut points were used only to define high-ETR and low-ETR groups within each method and were not intended for direct numerical comparison; high-ETR denotes values less than and equal to the median (more negative), and low-ETR denotes values greater than the median.

### Endpoints

The primary endpoint of the CROWN trial was PFS, as determined by blinded independent central review (BICR) per RECIST. These outcome reads were fully independent of all AI outputs. The cutoff date for this analysis is September 20, 2021, which corresponds to approximately 3 years of follow-up.^11^ The 3-year PFS by BICR, instead of 5-year by investigator,^6^ was used in this study so that we can evaluate the AI platform’s performance against blinded central read. The reliability of the AI platform was initially demonstrated by comparing its tumor diameter measurements with those obtained by 2 independent readers, ensuring a high level of concordance. Response assessment was further validated by comparing intracranial efficacy outcomes and best overall response discordance rates between the AI system and the human readers. Time-dependent outcomes, such as time to intracranial progression (iTTP), were evaluated to confirm that AI-derived measurements aligned with conventional assessments. Subsequently, we evaluated whether lesion-level ETR at the first post baseline imaging assessment is associated with PFS, independent of treatment arm.

### Statistical Analysis

As the primary endpoint of the study was met at the planned interim analysis, no further formal PFS analysis is planned, per protocol.^11^ This post hoc analysis was unplanned and conducted to explore the feasibility of AI-powered intracranial response assessment and identify new potential prognosticators. No updated overall survival (OS) analysis was performed at this time. The correlation and agreements between tumor assessment obtained by BICR and AI were assessed by Pearson’s correlation coefficient,^14^ 1-way ANOVA,^15^ and Cohen’s kappa coefficient for intracranial response categories.^16^ Kaplan-Meier methodology was applied to evaluate iTTP and PFS, with comparisons tested using the log-rank test.^17^ Statistical significance was set at *P* < .05.

To estimate the prognostic value of ETR independent of treatment, we fitted treatment-neutral Cox proportional-hazards models stratified by treatment arm (lorlatinib vs crizotinib). The clinical base model included the largest brain-met size (mm), number of BMs, and extracranial metastasis (yes/no). Early tumor response was represented binarily at prespecified cohort medians. We compared ETR models with the clinical base model using the likelihood-ratio χ². Overall discrimination was summarized by Uno’s concordance index (C-index) and time-dependent ROC area under the curve, [AUC(*t*)], with inverse-probability-of-censoring weighting (IPCW). Specifically, we reported the Δ*C* of *C* (Clinical + Target-ETR) vs *C* (Clinical + All-lesion-ETR). Uncertainty for *C* and Δ*C* was quantified by patient-level bootstraps (*B* = 1000), resampling individuals with replacement within each replicate. Dynamic AUC(*t*) with IPCW, according to the method proposed by Blanche et al,^18^ was evaluated on a prespecified grid from baseline to 36 months, corresponding to the cohort’s median follow-up. We summarized performance by the integrated AUC (0-36 months) and reported a single prespecified horizon of 12 months.

To contextualize treatment-arm effects, we additionally fit an ordinary (nonstratified) Cox model including treatment arm as a covariate alongside the clinical adjustments and binary ETR.

## Results

### Agreement and Validation of AI Measurement

**Figure 2.**
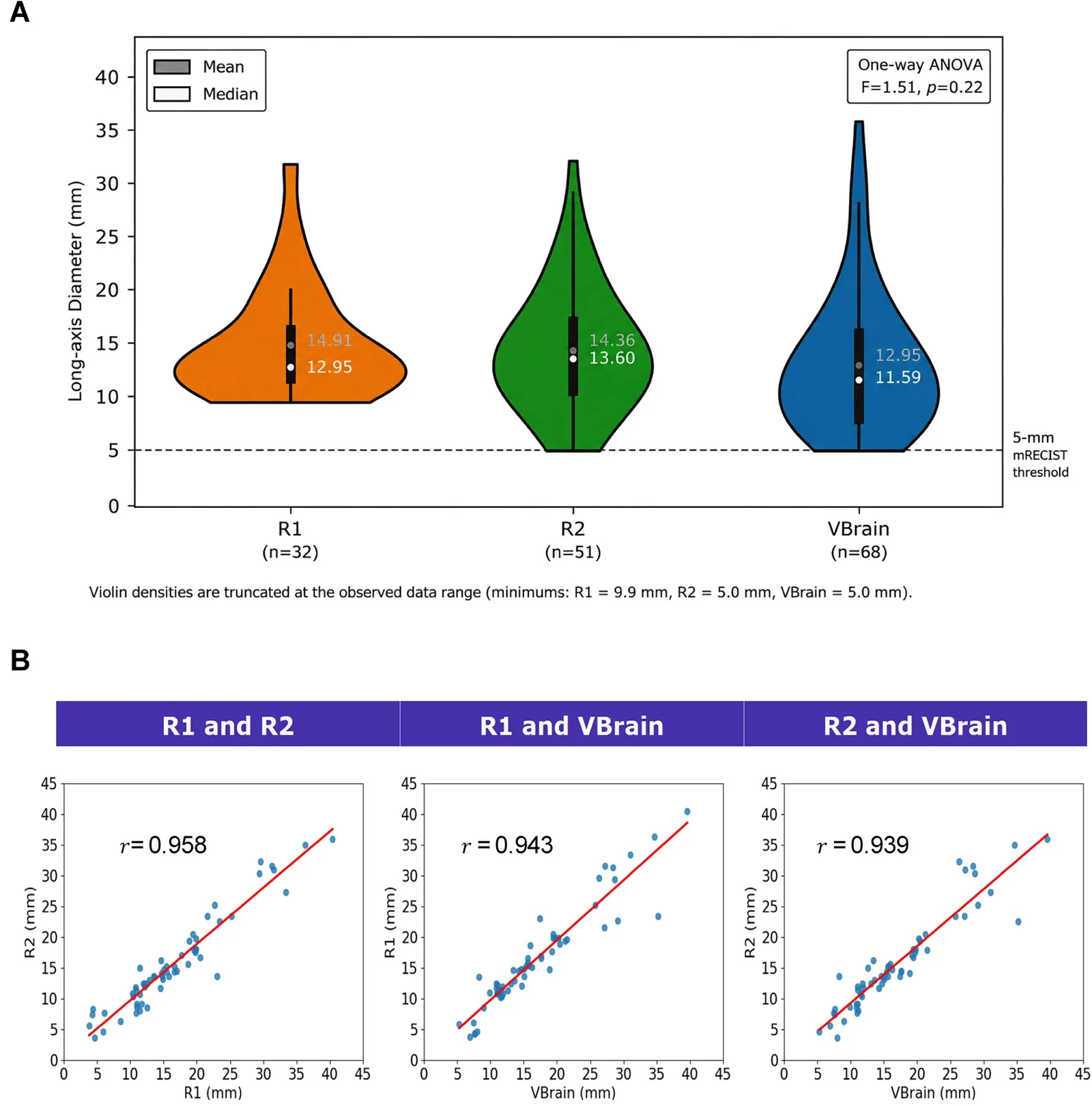
Baseline diameter distribution and agreement of mRECIST-eligible target lesions across readers. (A) Violin plots of baseline long-axis diameters (mm) for target lesions measured by blinded central readers R1 and R2 and by VBrain. For this mRECIST-based comparison, target lesions were required to be ≥5 mm. Violin densities were truncated at the observed data range to avoid kernel-density extrapolation beyond the minimum observed lesion diameter. White dot = median; gray dot = mean; thick bar = IQR; whiskers = range. Distributions were similar across readers (1-way ANOVA *F* = 1.51, *P* = .22). (B) Pairwise agreement between VBrain and each BICR reader at baseline. Scatterplots show per-lesion diameters with least-squares fit (solid line). Pearson correlations indicate high concordance: r = 0.943 (VBrain vs R1) and r = 0.939 (VBrain vs R2). Abbreviations: IQR, interquartile range; mRECIST, modified response evaluation criteria in solid tumors; R1/R2, central readers.

Seventy-two patients with baseline BMs comprised the analysis cohort (Figure 1). Baseline demographics, intracranial disease burden, and imaging intervals were broadly similar between lorlatinib and crizotinib cohorts (Table 1). Among these patients, the numbers of mRECIST-eligible target lesions selected at baseline were 32 by reader 1 (R1), 51 by reader 2 (R2), and 68 by VBrain, using the prespecified long-axis size criterion of ≥5 mm. VBrain therefore selected more target lesions than either human reader. However, the size distribution of VBrain-selected lesions was broadly comparable with that of reader-selected lesions and was not dominated by very small borderline lesions (Figure 2A). The median sizes of the target lesions measured were 11.6 mm for VBrain, 13.0 mm for R1, and 13.6 mm for R2, with no statistically significant difference in baseline diameter distribution across methods (1-way ANOVA *F* = 1.51, *P* = .22). Figure 2B demonstrates the correlation of measured diameters between VBrain and 2 BICR readings. The Pearson correlation coefficients showed a high correlation in target-lesion measurements between VBrain and the 2 readers (r = 0.943 and 0.939).

**Table 1.** Baseline characteristics and imaging schedule by treatment arm

	Lorlatinib (*n* = 32)	Crizotinib (*n* = 40)
A. Demographics and clinical features		
Age, years—median (IQR)	59.5 (19.8)	51.5 (23.0)
Female—*n* (%)	24 (75.0)	24 (60.0)
ECOG performance status		
0/1—*n* (%)	31 (96.9)	37 (92.5)
2—*n* (%)	1 (3.1)	3 (7.5)
Extracranial metastasis—*n* (%)	18 (56.2)	29 (72.5)
B. Intracranial disease burden at baseline		
Number of target lesions (R1)	21	11
Number of target lesions (R2)	32	19
Number of predicted lesions (AI)	251	143
Number of intracranial lesions—median (IQR) (AI)	3.0 (6.0)	1.5 (3.0)
Largest lesion long-axis diameter, mm—median (IQR) (AI)	12.9 (10.0)	8.5 (11.6)
C. Follow‑up imaging and timing		
Baseline → first on‑treatment scan (interval, days—median (IQR)	62.0 (14.0)	63.0 (13.5)

Target lesions (R1/R2) are human counts from 2 independent readers at baseline; predicted lesions (AI) are lesions detected by VBrain on baseline MRI. Intracranial lesion count and the largest lesion long-axis diameter are derived from the AI segmentation.

Abbreviations: AI, artificial intelligence; IQR, interquartile range.

### Intracranial Response Assessment

Among 666 time points for paired-response assessments, agreement between the 2 central readers was 64.3% (discordance 35.7%), with Cohen’s *κ *= 0.669 and weighted *κ* = 0.731/0.777 (linear/quadratic). Agreement between VBrain and each reader was comparable: 64.9% vs R1 (discordance 35.1%, *κ* = 0.623, weighted *κ* = 0.689/0.733) and 66.5% vs R2 (discordance 33.5%, *κ* = 0.650, weighted *κ* = 0.695/0.724). Most discrepancies were concentrated within adjacent categories (Tables S1 and S2). Overall, Vbrain-reader agreement falls within the range of human interreader variability, supporting the use of AI-derived assessments in subsequent analyses.

### Agreement of iTTP

Figure S2 demonstrates the iTTP derived from AI and human readers. The median iTTP in the crizotinib arm is 11.2, 9.4, and 9.5 months from VBrain and the 2 central readers, while the median iTTP is not reached in the lorlatinib arm or the whole participants. There is no significant difference by the log-rank test. The *P*-value by log-rank tests is 0.707 (R1 vs R2), 0.872 (R1 vs VBrain), and 0.840 (R2 vs VBrain).

### Prognostic Value of ETR

**Figure 3.**
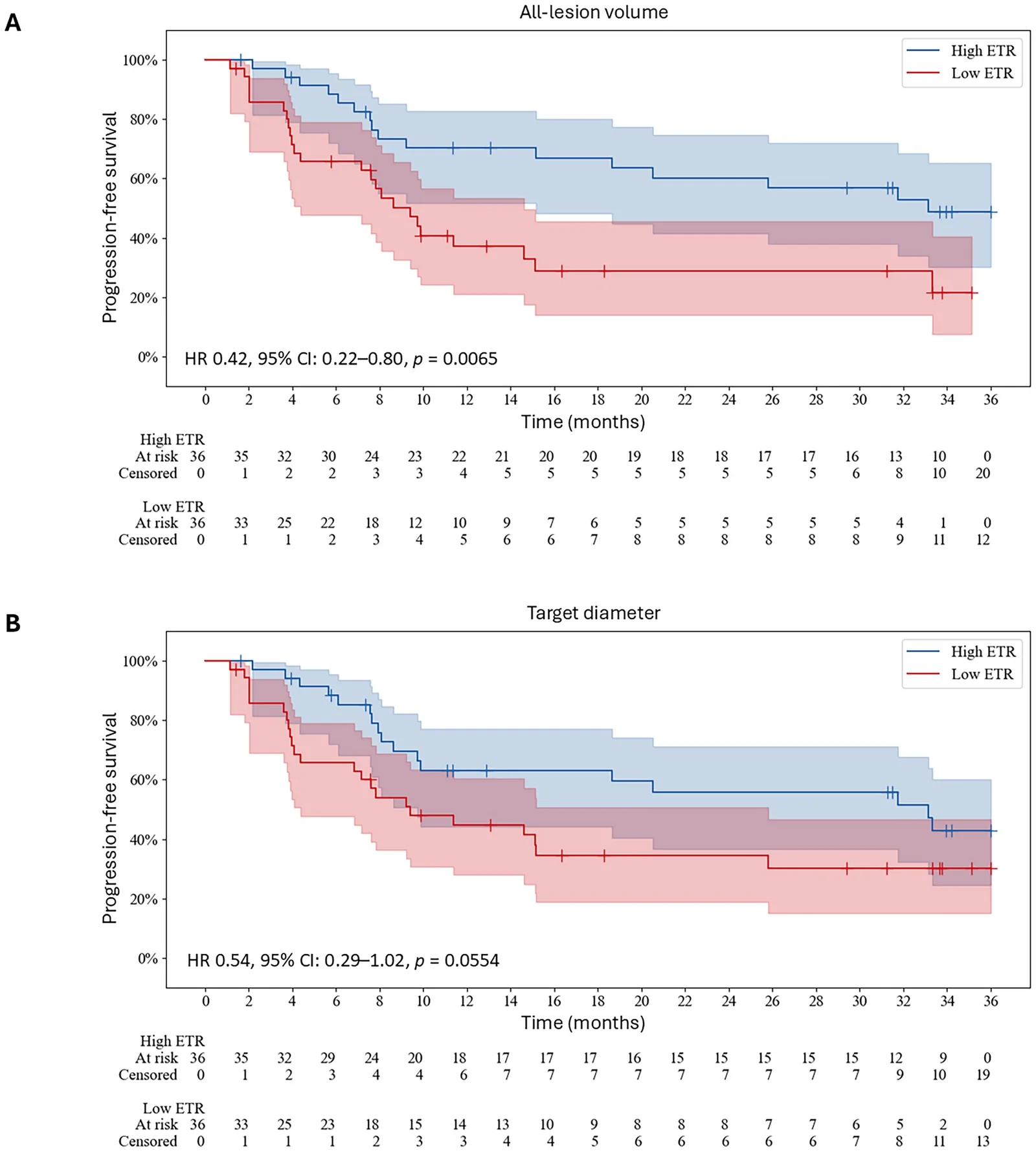
Kaplan-Meier analysis of systemic progression-free survival (PFS) stratified by early tumor response (ETR) at the first follow-up (FU1), combining the lorlatinib and crizotinib arms. (A) PFS curves based on all-lesion volumetric ETR, using the whole-cohort median (−67.9%) as the cutoff. (B) PFS curves based on RECIST-defined target-lesion diameter ETR, using the whole-cohort median (−24.2%) as the cutoff. Shaded areas represent the 95% CIs. High ETR indicates patients with greater tumor shrinkage than the median, while low ETR represents those with less shrinkage, regardless of treatment arms.

Beyond the target-lesion framework of mRECIST, VBrain tracked 322 tumors above the prespecified size threshold (≥2 mm). At OTS1 (ie the first postbaseline imaging assessment), AI volumetric ETR (all-lesion, median split) separated PFS more clearly than RECIST target-diameter response. In the unadjusted Cox analyses corresponding to the Kaplan-Meier comparisons, the HRs were 0.42 (95% CI, 0.22-0.80; P = .0065) for AI volumetric ETR and 0.54 (0.29-1.02; P = .055) for RECIST target-diameter ETR (Figure 3).

To evaluate the added value of incorporating ETR for predicting PFS, we constructed models stratified by treatment arm and adjusted for largest brain tumor size, number of BMs, and extracranial metastasis as the base model. In these adjusted models, AI-ETR (all-lesion volumetric change) remained independently associated with longer PFS (HR 0.44, 95% CI, 0.23-0.86; *P* = .0167), whereas target-diameter ETR showed a nonsignificant trend (HR 0.64, 95% CI, 0.29-1.41; P = .2654). Base clinical covariates were not significant in these OTS1-landmarked, treatment-neutral models (all *P*-values greater than .12) (Table 2; Figure S3).

**Table 2.** Incremental value of early tumor response (ETR) to treatment-stratified Cox models for PFS

Covariate	Base model HR (95% CI); *P*	+ Target ETR (diameter) HR (95% CI); *P*	+ AI-ETR (all-lesion volume) HR (95% CI); *P*
Extracranial metastasis (yes vs no)	1.71 (0.86 to 3.40); .125	1.46 (0.69 to 3.08); .319	1.69 (0.85 to 3.37); .136
Intracranial lesion count (per lesion)	0.98 (0.93 to 1.02); .305	0.99 (0.94 to 1.03); .530	0.99 (0.95 to 1.04); .668
Largest lesion diameter (per mm)	1.00 (0.96 to 1.04); .995	1.01 (0.97 to 1.05); .660	1.00 (0.96 to 1.03); .859
ETR—Target diameter (high vs low)	—	0.64 (0.29 to 1.41); .265	—
ETR—All-lesion volume (high vs low)	—	—	0.44 (0.23 to 0.86); .017

Model performance			
Metric	Base	+ Target ETR	+ AI-ETR
Likelihood-ratio χ² (overall)	3.99	5.24 (Δ + 1.25); *P *= .263	9.95 (Δ + 5.96); *P *=0.041
Δ*c*-index (95% CI)	—	0.0315 (−0.0178 to 0.1185)	0.0686 (0.0017 to 0.1564)
Mean AUC(*t*) gain, 0-36 months	0.5501	0.6280 (Δ + 0.0779)	0.7064 (Δ + 0.1563)

The clinical base model included extracranial metastasis, intracranial lesion count, and largest intracranial lesion diameter, with stratification by treatment arm. Augmented models additionally included either Target-ETR, based on RECIST target-lesion diameter, or AI-ETR, based on all-lesion intracranial volume. Each ETR metric was dichotomized at the cohort median as high vs low response. Hazard ratios reported with multivariable-adjusted estimates.

Abbreviations: AUC(*t*), time-dependent area under the ROC curve; ETR, early tumor response; HR, hazard ratio.

**Figure 4.**
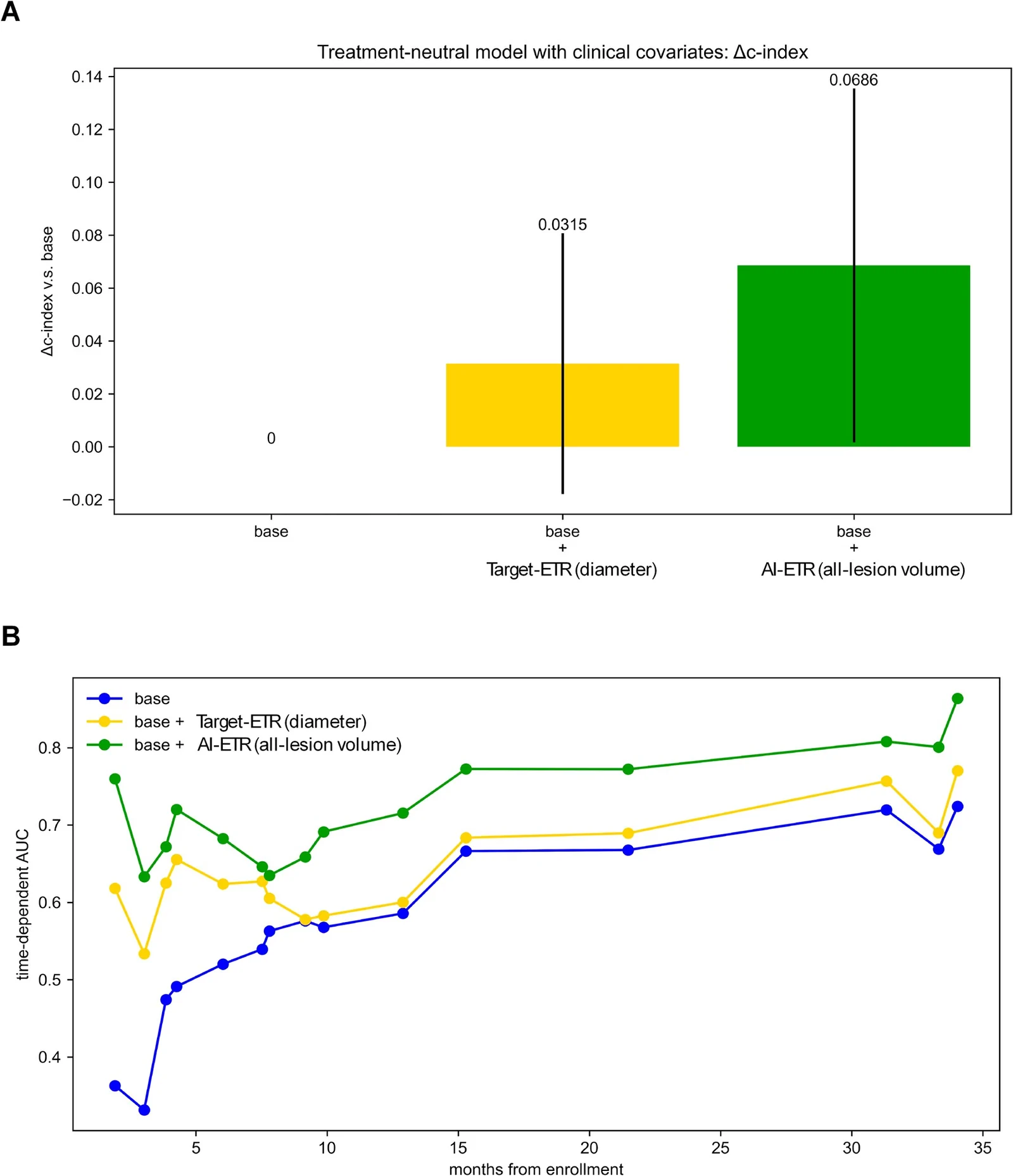
Incremental prognostic value of early intracranial response. (A) Change in concordance (Δ*c*-index) relative to the treatment-neutral clinical base model after adding target-ETR (RECIST target diameter) or AI-ETR (all-lesion volume). Gains were 0.0315 and 0.0686, respectively; vertical lines indicate bootstrap 95% CIs from Uno’s C (IPCW). The base model included extracranial metastasis, intracranial lesion count, and the largest intracranial lesion diameter and was stratified by treatment arm. (B)Time-dependent AUC(*t*) across 36 months for Base, Base + Target-ETR, and Base + AI-ETR; the AI-ETR curve remains highest throughout the follow-up. Mean AUC(*t*): 0.5501 (Base), 0.6280 (Base + Target-ETR), 0.7064 (Base + AI-ETR). Abbreviations: AUC(*t*), time-dependent area under the ROC curve; ETR, early tumor response; IPCW, inverse-probability-of-censoring weighting; OTS1, first on-treatment scan.

Relative to the base model, Δ*c*-index increased by +0.0686 (95% CI, 0.0017-0.1564) with AI-ETR and by 0.0315 (95% CI, −0.0178 to 0.1185) with target-ETR; the likelihood-ratio improvement was significant for AI-ETR (Δχ² +5.96, *P* = .0414) but not for target-ETR (Δχ² +1.25, P = .2632) (Figure 4A; Table 2). Time-dependent ROC analysis showed parallel gains in discrimination across the entire 3-year follow-up. The mean AUC(*t*) increased from 0.5501 (base) to 0.6280 with target-ETR (Δ = 0.0779) and to 0.7064 with AI-ETR (Δ = 0.1563) (Figure 4B). At 12 months, AUCs were 0.562 (base), 0.601 with Target-ETR (Δ = 0.039), and 0.684 with AI-ETR (Δ = 0.122), reinforcing the AI-ETR model’s superior discrimination (Figure S4).

### ETR Interaction With Treatment Arm as Prognostic Factor

Given that treatment arm (lorlatinib vs crizotinib) has been identified as the most significant prognostic factor (HR 0.21) for BM-positive ALK-NSCLC populations over a 3-year follow-up of the CROWN trial,^11^ its interaction with ETR was analyzed (Table S3). Treatment arm remained the dominant prognosticator, showing a statistically significant association with PFS across all models (HR 0.24-0.26, P < .005). By contrast, binary ETR did not reach statistical significance in any model (HR 0.78-0.89, P = 0.51-0.73). No evidence for a statistical interaction between ETR and treatment arm was observed. Exploratory analyses within each treatment arm showed consistent but nonsignificant trends favoring higher volumetric ETR with longer PFS, particularly in the lorlatinib arm (Figure S3).

## Discussion

In this exploratory analysis of the CROWN trial, we investigated the feasibility and prognostic significance of an AI-powered intracranial response assessment, with a particular focus on ETR in advanced ALK-positive NSCLC patients with BMs at baseline. Artificial intelligence-derived intracranial measurements and assessments showed reliability comparable to blinded central readers. At the first postbaseline assessment, AI all-lesion volumetric ETR separated PFS more clearly than the RECIST target-lesion diameter did. In treatment-neutral Cox models, AI-ETR was independently associated with a lower risk of progression, with a larger Δ*c*-index and higher AUC(*t*) through the 3-year follow-up, indicating better early discrimination of patients at risk of progression. No interaction between treatment and ETR was detected; AI all-lesion ETR stratified 3-year PFS in both arms, including the lorlatinib arm despite few events, with higher ETR indicating longer PFS.

The initial validation showed excellent agreement between the AI-derived lesion measurements and independent radiologists r = 0.94. We acknowledge that VBrain selected more mRECIST-eligible target lesions than either human reader (R1, 32; R2, 51; VBrain, 68). Importantly, these mRECIST-based target lesions were selected using the prespecified ≥5 mm criterion, and their size distribution was broadly comparable across VBrain and the 2 readers (Figure 2A), suggesting that the additional VBrain-selected target lesions were not predominantly very small borderline foci. This interpretation is further supported by prior validation of VBrain in BMs,^19^ which showed a low mean false-positive count and high lesionwise sensitivity, particularly for lesions ≥5 mm.

Target-lesion selection under RECIST 1.1 shows notable inter-/intraobserver variability and is a major source of between-reader disagreement in response assessment.^20^^,^^21^ Within the CROWN BICR, 35.7% of paired reads were discordant, well within the 30%-40% range commonly reported in multicenter oncology trials.^22-24^ The weighted *κ* values (0.73 linear; 0.78 quadratic) exceeded the unweighted *κ* (0.67), indicating that most disagreements reflected adjacent categories rather than major misclassifications. Agreement between AI and each reader was of the same order: 35.1% discordance vs R1 (*κ* = 0.623; weighted *κ* = 0.689/0.733) and 33.5% vs R2 (*κ* = 0.650; weighted *κ* = 0.695/0.724), again consistent with primarily adjacent-category differences. Despite this expected variability, intracranial efficacy endpoint of iTTP was concordant across AI- and reader-derived assessments, supporting the protocol compatibility of the AI method and its use in subsequent AI-derived ETR analyses.

In this study, early intracranial response quantified by AI volumetric all-lesion shrinkage at OTS1 provided a more informative prognostic signal than RECIST target-lesion diameter. In the unadjusted analyses corresponding to the Kaplan-Meier comparisons, high-shrinkage AI-ETR was associated with longer PFS (HR 0.42; 95% CI, 0.22-0.80; *P* = .0065), whereas the association for RECIST target-diameter ETR did not reach statistical significance. In the multivariable model stratified by treatment arm and adjusted for baseline clinical factors, AI-ETR remained independently associated with longer PFS (adjusted HR 0.44; 95% CI, 0.23-0.86). Adding AI-ETR to the clinical base model substantially improved discrimination over 0-36 months (mean AUC(*t*) 0.5501-0.7064; Δ*c*-index +0.0686). The incremental prognostic value of AI-ETR is clinically and biologically plausible. In ALK-positive NSCLC, intracranial and extracranial metastases are mostly driven by the same actionable ALK rearrangement, although additional site-specific resistance mechanisms and clonal evolution may occur.^25^ Early shrinkage of BMs may reflect broader tumor vulnerability to ALK inhibition. This interpretation is supported by CROWN, in which lorlatinib produced durable systemic PFS benefit together with robust intracranial efficacy.^5^^,^^6^ Because the CNS remains a pharmacologically challenging sanctuary site, AI-derived all-lesion volumetrics may provide an integrative imaging readout of effective drug exposure, total intracranial tumor burden, and interlesional response heterogeneity. Our findings suggest that AI-driven lesion-level assessment may serve as an early imaging biomarker for systemic disease control to enable early identification of low ETR, allowing clinicians to refine therapy sooner, trigger timely interventions, or consider alternative strategies in future prospective studies.

VBrain has been validated across multiple clinical settings and demonstrated a sensitivity of 96.2% in stereotactic radiosurgery (SRS) for BMs measuring ≥5 mm in diameter.^19^ Additionally, when integrated into the workflow for SRS, VBrain has been shown to significantly enhance interreader agreement, improve contouring accuracy, and increase efficiency.^26^ Using VBrain in metastatic NSCLC treated with osimertinib, mixed lesion response emerged as prognostic.^27^ More broadly, AI volumetric segmentation in neuro-oncology achieves high overlap with expert annotations.^28^ Automated RECIST target-lesion tracking has shown reliability comparable to radiologists (Fleiss’ κ ≈ 0.63),^29^ and whole-body pipelines can quantify total tumor burden longitudinally.^30^ To our knowledge, no study has yet quantified end-to-end time savings for deep-learning systems across a full RECIST workflow in clinical trials. Earlier semiautomated tools reported 15%-45% reductions in reading time.^31^^,^^32^ In the present study, end-to-end inference for intracranial segmentation and measurement was <2 min per brain MRI, suggesting operational efficiency for large imaging datasets without altering BICR adjudication procedures.

Previous studies have evaluated the prognostic implications of early tumor shrinkage assessed by RECIST in advanced NSCLC treated with EGFR TKIs or chemotherapy.^33-35^ Besides our own AI results, emerging evidence indicates that volumetric response metrics have proved more sensitive and prognostically informative than 1- or 2-dimentional measurements. In a multicenter cohort with 185 BMs, volumetric criteria doubled the number of evaluable lesions, detected progression a median 3.3 months earlier. They also separated recurrence-free survival more clearly than the response assessment in neuro-oncology criteria for brain metastases (RANO-BM), which uses a 1-dimensional measurement of each lesion’s longest axis (log-rank *P *< .001).^36^ Likewise, in the phase II CheckMate-204 trial, volumetric responders showed stronger correlations with intracranial PFS and OS than either RECIST or RANO-BM.^37^ These findings highlight the potential limitations of RECIST criteria, suggesting that AI-assisted, lesion-level volumetric response evaluation may more comprehensively capture treatment-induced changes, particularly in a population where intracranial progression is a major determinant of survival.

However, our study has several limitations. The current analysis was exploratory and post hoc, with a limited number of patients. The work does not validate AI-ETR as a surrogate endpoint for OS or treatment effect; it only evaluates prognostic association with PFS. Larger prospective studies will be needed to validate the clinical utility and generalizability of AI-driven volumetric response metrics. Additionally, standardization of volumetric response thresholds and further evaluation of AI’s performance across different imaging platforms and centers remain essential before widespread adoption.

Despite inherent limitations, the AI platform demonstrated measurement reliability and response classification accuracy comparable to expert human readers. Future prospective studies should further explore the clinical integration of volumetric ETR as a supplementary prognostic marker to inform early therapeutic decisions in clinical practice.

## Conclusion

This study shows that an FDA-cleared clinical AI system can function as a digital endpoint tool within a global phase III oncology trial, enabling lesion-level volumetric assessment of intracranial disease that would be infeasible with manual workflows. While showing high-concordance with BICR assessment, digitized lesion-level volumetric ETR provided additional, clinically meaningful information beyond traditional RECIST criteria, suggesting its potential as an early surrogate marker for systemic efficacy. Further validation in larger prospective studies is necessary to confirm these findings and facilitate clinical adoption.

## Supplementary Material

vdag236_Supplementary_Data

## Data Availability

Upon request, and subject to review, Pfizer will provide the data that support the findings of this study. Subject to certain criteria, conditions, and exceptions, Pfizer may also provide access to the related individual deidentified participant data. See https://www.pfizer.com/science/clinical-trials/trial-data-and-results for more information.
