# Supplementary material for: Artificial intelligence-enabled early intracranial volumetric response predicts systemic progression-free survival in the phase III CROWN study
Source: Neurooncol Adv. 2026 Sep 7;8(1):vdag236. doi: 10.1093/noajnl/vdag236 (PMC13599776; doi:10.1093/noajnl/vdag236)
Supplement: Supplementary file 1 [file vdag236_supplementary_data.docx]

**Supplementary Figure S1. Early tumor response (ETR) following treatment initiation and its association with progression-free survival.**

Tumor burden was either measured by diameter (cm) or volume (cm^3^). Illustration of the reduction in tumor burden following treatment initiation, with ETR defined as the change observed at the first on-treatment scan (indicated by the arrow). The x-axis represents time in months, while the y-axis denotes the tumor burden. The impact of ETR on systemic progression-free survival (PFS) was further evaluated.

**Supplementary Figure S2. Kaplan-Meier analysis of intracranial time to progression (iTTP) based on assessments by two independent readers (R1 and R2) and the AI platform (VBrain).**

**The survival curves represent intracranial progression-free survival as determined by each method. There was no significant difference in iTTP between R1 and R2 (**$p$ **= 0.7069), R1 and VBrain (**$p$ **= 0.8719), or R2 and VBrain (**$p$ **= 0.8403), as assessed by the log-rank test.**

**Supplementary Figure S3. Multivariable treatment-neutral models for PFS**

Forest plots show hazard ratios (points) with 95% CIs (horizontal lines); the dashed vertical line marks HR=1. (A) Clinical base model: intracranial brain-metastasis (BM) number (per lesion), largest intracranial lesion long-axis diameter (per mm), and extracranial metastasis (yes vs no). (B) Base + Target-ETR (RECIST target-diameter change, high vs low). (C) Base + AI-ETR (all-lesion volume change, high vs low). Models are Cox proportional hazards stratified by treatment arm; two-sided Wald *p* values are shown. HR<1 favors longer PFS. *Abbreviations:* BM, brain metastasis; ETR, early tumor response.

**Supplementary Figure S4. Time-dependent ROC at 12 months**

Time-dependent ROC curves at 12 months comparing discrimination of the clinical base model (blue), base **+ Target-ETR** (orange), and base + **AI-ETR (all-lesion volume)** (green). Area under the curve (AUC) values were **0.562** (Base), **0.601** (Base+Target-ETR), and **0.684** (Base+AI-ETR). *Abbreviations:* ETR, early tumor response.

**Supplementary Figure S5. Progression-free survival stratified by early tumor response (ETR) using arm-specific median values within lorlatinib and crizotinib treatment arms.**

**We further evaluated ETR separately within each treatment arm by establishing arm-specific median ETR values as cut-points. In the lorlatinib arm, neither ETR assessed by target lesion diameter (HR 0.33; 95% CI, 0.07–1.64;** $p$ **= 0.154) nor by all-lesion volumetric measurements (HR 0.31; 95% CI, 0.06–1.52;** $p$ **= 0.125) reached statistical significance, likely due to the limited number of progression events. However, a trend favoring patients with higher ETR was observed, particularly notable in the all-lesion volumetric assessment (Figure S5A). In the crizotinib arm, the volumetric approach for all lesions (HR 0.49; 95% CI, 0.23–1.04;** $p$ **= 0.058) showed a clearer association with PFS compared to the target lesion diameter approach (HR 0.86; 95% CI, 0.41–1.82;** $p$ **= 0.698; Figure S5B). Taken together, these results suggest a consistent trend favoring the all-lesion volumetric approach in both treatment arms, despite the limited number of progression events in the lorlatinib group.**

**Kaplan-Meier curves represent PFS according to the ETR assessed either by all-lesion volumetric (left panel) or by target lesion diameter changes (right panel). In the lorlatinib arm (A), median ETR values were -65.60% (diameter-based) and -99.74% (volume-based). In the crizotinib arm (B), median ETR values were -5.13% (diameter-based) and -37.88% (volume-based).**

**Supplementary Table S1. Discordance rates in response assessment between independent readers and the AI platform.**

**The table summarizes the percentage of discordance observed in best overall response and time-point response assessments among Reader 1 (R1), Reader 2 (R2), and the VBrain AI platform.**

|  | **% of discordance R1 vs R2** | **% of discordance R1 vs VBrain** | **% of discordance R2 vs VBrain** |
| --- | --- | --- | --- |
| Best of Response | 37.5% (27 / 72) | 37.5% (27 / 72) | 34.7% (25 / 72) |
| Time Point Response | 35.7% (238 / 666) | 35.1% (234 / 666) | 33.5% (223 / 666) |

**Supplementary Table S2. Pairwise agreement in overall intracranial response categories**

Counts (CR, PR, SD, PD, NED) for R1 vs R2, R1 vs VBrain, and R2 vs VBrain. Agreement is summarized with *κ* (Cohen’s), linear‑weighted *κ*, and quadratic‑weighted *κ* for ordered categories (PD<SD<PR<CR<NED).

**A. R1 vs R2**

|  | CR | PR | SD | PD | NED |
| --- | --- | --- | --- | --- | --- |
| CR | 267 | 11 | 13 | 1 | 47 |
| PR | 5 | 16 | 0 | 0 | 2 |
| SD | 33 | 3 | 85 | 22 | 13 |
| PD | 5 | 1 | 2 | 41 | 4 |
| NED | 34 | 6 | 32 | 4 | 19 |

***κ*=0.669; linear‑weighted *κ*=0.731; quadratic‑weighted *κ*=0.777**

**B. R1 vs VBrain**

|  | CR | PR | SD | PD | NED |
| --- | --- | --- | --- | --- | --- |
| CR | 292 | 9 | 11 | 8 | 19 |
| PR | 8 | 12 | 0 | 3 | 0 |
| SD | 32 | 1 | 76 | 15 | 32 |
| PD | 4 | 0 | 14 | 32 | 3 |
| NED | 58 | 2 | 11 | 4 | 20 |

***κ*=0.623; linear‑weighted *κ*=0.689; quadratic‑weighted *κ*=0.733**

**C. R2 vs VBrain**

|  | CR | PR | SD | PD | NED |
| --- | --- | --- | --- | --- | --- |
| CR | 305 | 6 | 16 | 6 | 11 |
| PR | 9 | 17 | 0 | 8 | 3 |
| SD | 16 | 1 | 71 | 6 | 38 |
| PD | 11 | 0 | 19 | 33 | 5 |
| NED | 53 | 0 | 6 | 9 | 17 |

***κ*=0.650; linear‑weighted *κ*=0.695; quadratic‑weighted *κ*=0.724**

*Abbreviations: CR, complete response; PR, partial response; SD, stable disease; PD, progressive disease; NED, no evidence of disease.*

**Supplementary Table S3. Multivariable analysis incorporating early tumor response (ETR) and treatment arm as prognostic factors for progression-free survival (PFS).**

**Patients were categorized by median ETR, assessed using RECIST target lesion diameter, and all-lesion volume at the first on-treatment scan. Cox proportional hazard models were applied to estimate the hazard ratio (HR) and 95% confidence intervals (CI) for progression or death.**

| **Subgroup** | **Hazard Ratio  (95% CI)** | | ***p*-value** | **Likelihood Ratio Test** |
| --- | --- | --- | --- | --- |
| **Target-ETR (Diameter)** | | | | |
| ETR | | 0.89 (0.45, 1.75) | 0.73 | 17.94 on 2 df |
| Treatment ARM | | 0.24 (0.11, 0.53) | <0.005 |  |
| **AI-ETR (All lesion volume)** | | | | |
| ETR | 0.78 (0.37, 1.62) | | 0.51 | 18.27 on 2 df |
| Treatment ARM | 0.26 (0.11, 0.59) | | <0.005 |  |
